# Cook County Hospital

**Published:** 1883-09

**Authors:** E. P. Davis, C. E. Currie


### Hospital Reports.

### Article VI.

Cook County Hospital Reports. Reported by Drs. E. P Davis and C, E. Currie. Attending Physician, Dr. McWilliams.

Name, A. L., civil state M., Race W., age 40, occupation laborer, nativity german, diagnosis insolation. Admitted July 3, 1883, hour 4:50 p. m, discharged July 5, 1883, condition recovered. Service of Drs. McWilliams and Buchan, C. E. Currie, House Physician.

While at work, patient suddenly fell unconscious, and was taken to the hospital by police patrol wagon.

On his arrival at 4:50 p. m. his temperature was 107·5° axillary. Face pale, lips and finger tips bluish in color, pupils contracted, respiration stertorous; the surface of the body felt intensely hot; bowels had moved involuntarily.

He was placed in a wet pack with ice in the axillæ, groins, and at the back of neck. Pieces of ice were then rubbed outside of wet sheet over the abdomen and chest.

5 p. m., 108° rectal. Gave a large enema of ice water morphiæ S gr. $\frac{1}{3}$ hypodermically. Gave spts. frumenti hypodermically as his extremities became cool.

6:30 p. m. Patient had a tonic spasm and remained in opisthotonos for about three hours. The muscles of his jaws were firmly contracted. Constant twitching of the eyelids. Patient was removed from pack and placed in bed.

July 3, 7:00 p. m., 102·5° axillary. Gave quiniæ bisulph. grs. vi. hypodermically.

9:30 P. M., 104·5° rectal. Placed in wet pack and applied an ice-cap. Patient to be removed from pack when temperature fell to 102°.

11:30 P. M., 102·8.

July 4, 1:30 A. M., 101·5°. Patient was taken from the pack and the ice-cap removed.

6:30 A. M., 74, 99·2°. Patient seems perfectly conscious, but will not answer questions. Pupils normal; respiration normal; is perspiring freely. ℞. potass. bromide grs. xx., t. i. d.

4 P. M., 72, 98·6°. Watch temperature.

5 A. M., 72, 99°. Patient is perfectly conscious and talks rationally. Recollects nothing which happened July 3. Discharged recovered.

---

ATTENDING PHYSICIAN, DR. BUCHAN.

Name, G. J., civil state S., race W, age 25, nativity Sweden, diagnosis insolation, admitted July 6, 1883, hour 9:25 P. M., discharged July 9, 1883, condition recovered.

Patient had received treatment before he was brought to the hospital. On admission he was in a wet sheet with pieces of ice about him. Extremities cold, pulse weak, temperature 104·5° rectal. Hypodermics of spts. frument. and morph. sulph. Muscular twitchings and tonic contraction of muscles of neck and back.

Patient was placed in a wet pack until temperature was 102°. Ice-cap was allowed to remain during the night and the next day.

July 7, A. M., 104, 101·4°. Patient has taken nourishment and answers questions rationally.

2:00 P. M., 80, 98·6°.

8:00 P. M., 80, 97·6 axillary. Complains of cephalalgia, potass bromide grs. xxx.. Remove ice-cap.

July 8, A. M., 88, 98·4°. Slight vertigo on attempting to walk; sal Rochelle ℥s.

7:08 P. M. 62, 99°.

7:09 A. M., 76, 98·4°. Discharged recovered.

*Belladonna Poisoning from Atropia.*—In the case of a boy aged 15, a solution of atropia, grs. iv. to the ℥, was used for keratitis, two drops were put in the eyes every five minutes for half an hour to secure thorough dilatation of the pupils. The application was made in the morning, and no constitutional effect was observed until early the following morning, when the patient became excitable, and very apprehensive of violence. He grew better at daybreak, and said that he had been threatened by an adult patient in the ward, whose bed he had shared during the night.

The dilatation of the pupils was maintained by atropia used sparingly during the day, the patient appearing as usual.

At evening well marked delirium occurred; the eyes were brilliant, the throat somewhat dry; the delirium was busy, ecstatic, and, when the patient was not restrained, happy. Restraint was finally necessary, and under the use of morphia hypodermically, the patient speedily recovered. Withdrawal of the atropia and its subsequent use in weakened solution were followed by no return of the delirium.

*Tetanus Traumatic—Nerve Stretching.*—A colored man, very muscular, on June 19, jumped upon a pile of boards, running a rusty nail half an inch into the sole of his foot. The wound became swollen and painful, and in a week after the reception of the injury, stiffness of the muscles of the neck was felt. On admission, trismus was well marked, opisthotonos followed, with convulsions. June 24, the great sciatic nerve of the affected leg was exposed and stretched by the house surgeon; temporary relief only followed.

Convulsions increased in frequency and severity; the temperature rose, and after an unusually severe spasm, the patient died July 2.

*Chronic Cystitis, Cystotomy.*—In a patient who had suffered for over six months with cystitis, incision into the bladder in the median line of the perineum was made; the finger was introduced, and the bladder explored. Its walls were found thickened, but lacculated stone was discovered.

Mild injections were employed, and free drainage maintained through the incision. The patient was greatly benefited.

